# Metabolic phenotypes in a Lyz2Cre recombinase mouse model

**DOI:** 10.3389/fimmu.2025.1499858

**Published:** 2025-03-18

**Authors:** S. M. Niazur Rahman, Justin Hou Ming Yung, Allen Volchuk, Neil M. Goldenberg, Adria Giacca

**Affiliations:** ^1^ Department of Physiology, Faculty of Medicine, University of Toronto, Toronto, ON, Canada; ^2^ Program in Cell Biology, The Hospital for Sick Children, Toronto, ON, Canada; ^3^ Anesthesia and Pain Medicine, The Hospital for Sick Children, Toronto, ON, Canada; ^4^ Department of Anesthesiology, Faculty of Medicine, University of Toronto, Toronto, ON, Canada; ^5^ Department of Medicine, Faculty of Medicine, University of Toronto, Toronto, ON, Canada; ^6^ Institute of Medical Science, Faculty of Medicine, University of Toronto, Toronto, ON, Canada; ^7^ Banting and Best Diabetes Centre, University of Toronto, Toronto, ON, Canada

**Keywords:** Cre-Lox P system, Lyz2Cre, macrophage, glucose metabolism, insulin resistance, β-cell dysfunction, obesity-associated diabetes

## Abstract

The Cre-Lox system is essential in biomedical research for precise gene deletion in specific cell types, crucial for understanding genetic roles in disease. Although generally considered non-detrimental, Cre recombinase expression has been associated with potential adverse effects, including Cre toxicity, ectopic expression, and disruption of endogenous genes. We investigated the role of macrophage nucleotide-binding oligomerization domain (Nod1) in obesity-associated diabetes using myeloid-specific Nod1-knockout mice (Nod1 floxed crossed with Lyz2Cre). Our study examined Lyz2Cre as well as floxed control mice separately, unlike most research. Results indicated that Lyz2Cre expression alone impacts glucose metabolism, challenging the notion that Cre expression is harmless. This finding highlights the critical importance of including Cre-only controls in studies using floxed alleles to generate conditional knockout mouse models in order to ensure robust and accurate conclusions in molecular research.

## Introduction

Using the Cre-Lox system is the predominant approach in biomedical research to understand genetic and molecular signaling pathways. This system enables the precise and targeted deletion of almost any mouse gene in specific cell types, which is essential for gaining insights into the role of genes in disease processes. Cre recombinase selectively identifies specific DNA sequences called loxP (locus of x-over, P1) sites. Upon binding to the loxP sites, Cre can excise or invert the DNA segment, creating a tissue-specific knockout when driven by a tissue-specific promoter (1, 2). While it has been widely believed that Cre expression does not have detrimental effects, there have been reports suggesting potential untoward effects associated with Cre (3). In the absence of targeted gene loxP sites, fibroblasts (3) and pancreatic β cells (4) demonstrated undesired phenotypes only by Cre expression, leading to adverse effects on cellular physiology. Cre toxicity has been reported to impair angiogenesis (5), reduce blood cell counts (6), and lead to heart failure (7). Cre activation alone has been shown to induce primary lymphoma regression (8). Both constitutive Cre and inducible CreER have been shown to negatively impact mammalian cell health. Furthermore, the wide range of affected cell types suggests that Cre/CreER toxicity may be a general phenomenon across most mammalian cell types (9). Several studies have linked Cre expression to genotoxic effects (10, 11). These effects arise from recombination at cryptic loxP sites (12), disruption of endogenous genes (13), and off-target DNA cleavage (10). Cre protein levels may influence the severity of toxicity (14). The lysozyme 2 (Lyz2) gene is widely expressed in myeloblasts, macrophages, and neutrophils, making it a widely used marker for myeloid cells in mice (15). However, Lys2-driven recombination unexpectedly showed reporter protein expression in non-myeloid cells, including type II lung alveolar cells (16) and some other cells (17, 18), including neurons of several brain regions (19), which may affect metabolic function. It is well-known that Cre insertion in the Lyz2Cre mouse available from Jackson lab deletes the endogenous gene (4, 20), which is critical for the function of myeloid cells (21). Our laboratory has studied the role of an immune receptor, nucleotide-binding oligomerization domain 1 (Nod1), in the context of obesity associated diabetes (22). Furthermore, the transplantation of bone marrow of whole body Nod1-null mice was reported to protect against high-fat diet-induced insulin resistance (23). We wished to investigate the effect of the deletion of this receptor in myeloid cells on lipid-induced pancreatic β-cell dysfunction (β-cell lipotoxicity) (24). We unexpectedly observed that Cre Lysozyme 2 (Lyz2) expression impacts metabolic function, even in the absence of lox-P sites, i.e., without deletion of Nod1.

## Methods

### Animals

All procedures were in accordance with the Canadian Council of Animal Care Standards and were approved by the Animal Care Committee of the University of Toronto with the study protocol number 20011526. The mice used for all experiments were male and 12 to 14 weeks old. Ear clips were obtained for genotyping purposes. Genotyping was conducted using the Wisent Genotyping Kit, and DNA was extracted according to the manufacturer’s protocols. The PCR primers and representative micrographs of genotyping results are shown in **Supplementary Figure 1**. Myeloid-specific Nod1-knockout (KO) mice were generated by crossing Nod1 floxed mice on a C57BL/6 background obtained from Dr. Dana J. Philpott (25) with mice expressing lysozyme 2 (Lyz2) Cre (Jackson Laboratory #004781) also on a C57BL/6 background. Nod1 floxed (Flox) and Lyz2Cre (Cre) mice were used as controls. Both controls were either littermates or mice from the same breeding colony. For all experiments, heterozygous Lyz2Cre mice were used.

All animals were housed in the University of Toronto’s Department of Comparative Medicine (DCM) facility, exposed to a 12-hour light-dark cycle, and had free access to water and standard rodent chow, which contained 58% carbohydrate, 24% protein, and 18% fat by calories (Harlan Tekland #2018).

### Mouse surgery

Mice were anesthetized using isoflurane (3%–3.5% for induction, 1.5%–2% for maintenance). Analgesics (Buprenorphine SR, 1 mg/kg) were administered pre-operatively to maintain therapeutic serum levels during recovery. A cannula made from 10–11 cm of polyethylene tubing (PE-10) and 1.5 cm of silastic tubing was inserted into the jugular vein, reaching the right atrium. The silastic tubing was tapered for easier insertion. The cannula was flushed with heparinized saline (40 units/mL) to prevent clotting, exteriorized at the back of the neck, and secured with tape. The mice were then placed in individual cages and allowed to recover for 3–5 days with free access to food and water. After surgery, the mice were continuously monitored until they regained consciousness and were fully ambulatory. Post-operative monitoring was conducted twice daily by both the surgeon and the DCM veterinarian to check for signs of distress or infection. A detailed log was maintained to document animal behavior, incision sites, and cannula condition.

### Infusion treatment

Our model for inducing β-cell lipotoxicity through prolonged elevation of plasma palmitate levels *in vivo* involves infusing mice with ethyl palmitate (PAL) at a rate of 0.12 μmol/min for 48 hours (26). Direct infusion of unbound palmitate is highly toxic due to its detergent effects; however, adding an ethyl group to the carboxylic end of palmitate neutralizes this toxicity. Ethylpalmitate is hydrolyzed into ethanol and palmitate by ethyl esterases in mouse plasma (26), therefore, ethanol served as the vehicle control (VEH). Mice were briefly anesthetized with 1.5% isoflurane to connect the inserted catheter to our infusion setup, which allowed them full mobility in their cages. We used syringe pumps (model # PHD2000) from Harvard Apparatus Inc. for the infusions. Throughout the 48-hour infusion, the mice had access to food and water *ad libitum*. After the infusion period, a hyperglycemic clamp was conducted to assess *in vivo* β-cell function.

### Hyperglycemic clamp

The mice were fasted for 5 hours prior to the start of the clamp, after which the mice were moved into the restrainer for the duration of the experiment. Blood glucose was elevated and maintained at ~20mM by an intravenous glucose infusion. A 37.5% glucose solution was used as the infusate to achieve the hyperglycemic target while minimizing the infusion volume, and it was co-infused with ethylpalmitate or vehicle using a 2-way connector. Blood glucose was measured every 10 minutes during the 2-hour clamp via tail vein blood sampling, and the glucose infusion rate (Ginf) was adjusted accordingly. A higher Ginf represents better glucose tolerance, which is determined by increased ß-cell secretion and decreased insulin clearance to compensate for palmitate-induced insulin resistance. At time points 0, 100, and 120 minutes, 50–100μL of plasma was collected from the tail vein to measure insulin and C-peptide levels.

### Plasma glucose, insulin, C-peptide, and free fatty acid measurements

Mouse plasma glucose concentrations were measured using the HemoCue Glucose 201 Analyzer.

Plasma obtained at times 0, 100, and 120 min of the clamp was used to determine levels of insulin and C-peptide. These samples were analyzed by enzyme-linked immunosorbent assays that are sandwich-type immunoassays obtained from ALPCO (Catalog #80-INSMS-E01, E10 for insulin, Catalog #80-CPTMS-E01 for C-peptide). Standard solutions were run in duplicate, while samples were run in singlicate. Both inter- and intra-assay variations are below 5% for the insulin assay and below 7% for the C-peptide assay. The insulin assay had a sensitivity of 10.34 pM with a detection range of 32.41 to 61,189.66 pM, while the C-peptide assay had a sensitivity of 7.6 pM with a detection range of 60 to 3,000 pM. Basal plasma (0 min) was used for free fatty acid (FFA) determination. The samples were analyzed by an enzymatic colorimetric assay obtained from FUJIFILM Wako Diagnostics (Catalog #999-34691, #995-34791, #991-34891, #993-35191). Standard solutions and samples were run in duplicate as per the manufacturer’s protocol. The enzymatic reaction generated a purple pigment, quantified at 550 nm using a microplate reader. FFA concentrations were determined using a standard curve, with a detection range of 0.07–2.50 mEq/L and a sensitivity of 0.04 mEq/L.

### Calculations

#### Insulin clearance index

The steady state molar ratio of plasma C-peptide to plasma insulin was taken as an established index of insulin clearance (27), which is extensively used in mice (28). This index represents insulin removal from plasma by the liver. The conversion from proinsulin to insulin in ß cells generates C-peptide, and insulin and C-peptide are co-secreted in response to glucose, but insulin is cleared by the liver in a regulated fashion, whereas C-peptide is not. The concentrations of insulin and C-peptide in plasma were measured during time points 100 and 120 of the clamps.

#### Insulin sensitivity index

Insulin sensitivity during the hyperglycemic clamp was calculated as the Ginf/plasma insulin at the steady state (the last 20 minutes of the clamp). With the hyperglycemic clamp, it is possible to obtain indices of both insulin sensitivity and ß-cell function, although the sensitivity index (SI) has limitations at high insulin levels due to the non-linearity of insulin kinetics (29).

#### Disposition index

The disposition index (DI) is a measure of ß-cell secretory function in the context of the insulin sensitivity of the subject. Normal ß cells are able to compensate for insulin resistance by increasing insulin secretion, and therefore, ß-cell secretory function *in vivo* must be evaluated considering insulin sensitivity. DI, which corresponds to Ginf, barring changes to insulin clearance, is the best-established index of ß-cell function *in vivo* (30). In the present study, DI was calculated by multiplying C-peptide as an index of insulin secretion by SI as we previously published (31).

### Phagocytosis assay

Bone marrow-derived macrophages (BMDMs) were isolated from the femurs and tibiae of untreated Nod1flox, Lyz2Cre, and Nod1-KO mice. A standard Fc receptor-mediated phagocytosis assay was performed as described previously (32, 33). Medium-sized (5 µm) microspheres were opsonized with human immunoglobulin G (IgG) and incubated with differentiated macrophages for 30 minutes at 37°C. After incubation, cells were fixed with paraformaldehyde (PFA) and labeled with Alexa647-conjugated anti-human IgG to stain external (non-phagocytosed) microspheres. The cells were then washed, permeabilized with 0.1% Triton X-100 for 5 minutes, and labeled with Alexa488-conjugated anti-human IgG to identify internalized microspheres. DAPI staining was used for nuclear visualization, and images were acquired using a spinning disk confocal microscope (4–5 fields per condition). Internalized microspheres appeared green, while external microspheres were purple. Quantification was performed by counting the number of internalized microspheres in 50 cells per condition.

### Statistical analysis

Data are represented as mean ± SEM. Given a coefficient of variation of 32% in our primary endpoint, i.e., Ginf, and an effect size of 68%, an *n* ≥ 4/group gave us a power ≥ 0.8 with 2*α*=0.05. Statistical differences were evaluated with one-way ANOVA and the Holm–Sidak *post hoc* test for multiple comparisons. P<0.05 was considered significant. The analysis was performed using GraphPad Prism 9.

## Results

### Lyz2Cre protects against palmitate-induced glucose intolerance similar to Nod1 knockout

**Figure 1:**
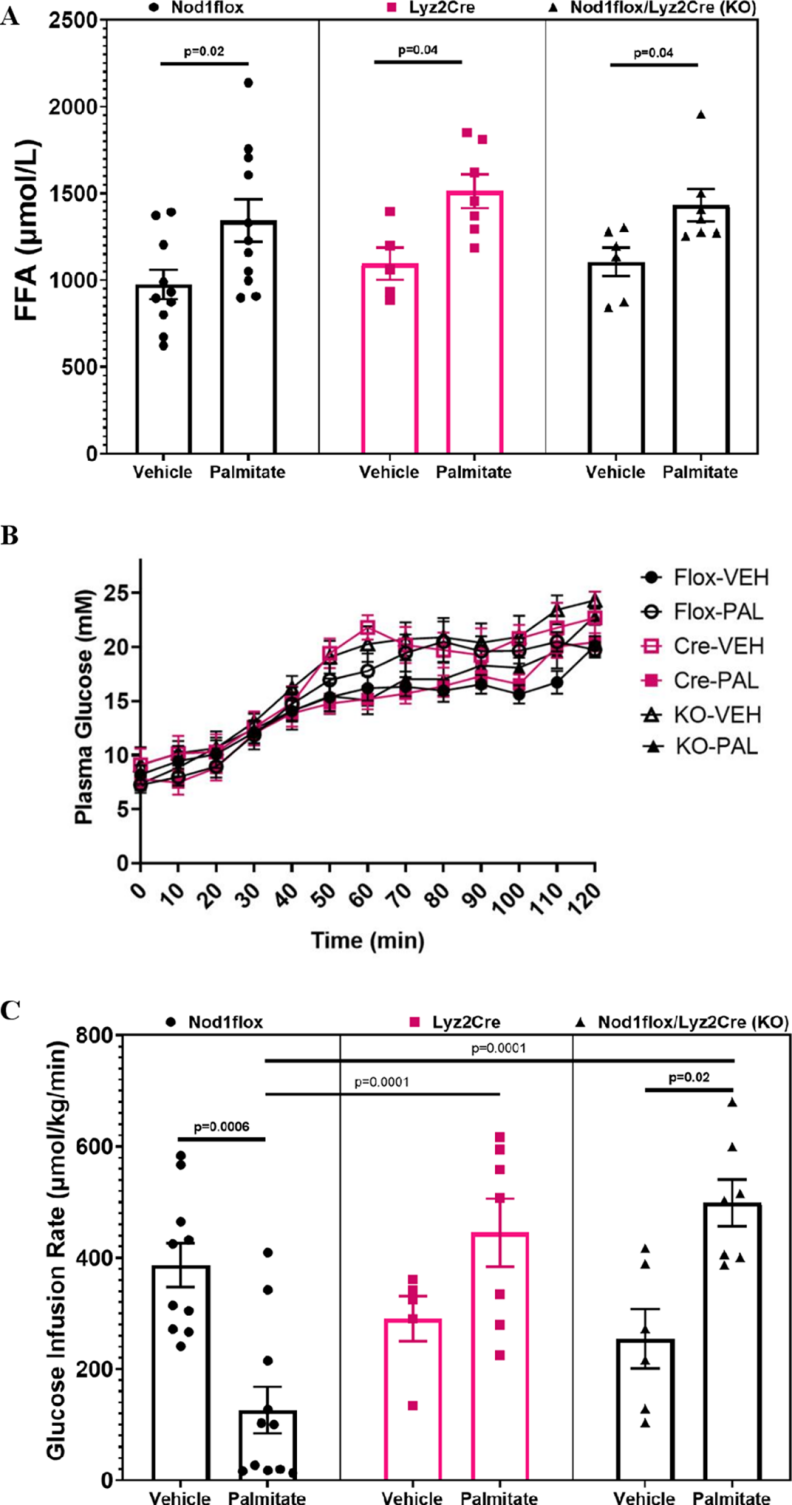
Basal plasma free fatty acids (FFAs) **(A)**, plasma glucose **(B)**, and glucose infusion rates **(C)** during hyperglycemic clamps after a 48-h infusion of either ethanol vehicle or ethylpalmitate in 12 to 14-week-old male mice. Nod1flox: Vehicle (Flox-VEH) (n=10), Palmitate (Flox-PAL) (n=11); Lyz2Cre: Vehicle (Cre-VEH) (n=5), Palmitate (Cre-PAL) (n=7); Nod1flox/Lyz2Cre (KO): Vehicle (KO-VEH) (n=6), Palmitate (KO-PAL) (n=7). Results are mean ± SEM. One-way ANOVA: **(A)** p=0.003; **(C)** p=0.0001. Significant differences between groups obtained with the Holm–Sidak test are shown in the graphs.

Following 48 hours of infusion, plasma FFAs were significantly increased in PAL-infused mice compared to the respective VEH-treated groups (**Figure 1A**). Plasma glucose levels increased to ~20mM in all groups during the last 20 min of the clamp (**Figure 1B**). The Ginf necessary to achieve and maintain the hyperglycemic target is a measure of glucose tolerance. As expected, the flox control PAL mice but not the Nod1-KO mice had reduced Ginf compared to VEH (**Figure 1C**); however, Ginf was not reduced with PAL in the Cre control group. This suggests that myeloid Cre expression alone was enough to induce an anti-inflammatory phenotype, thus protecting against palmitate-induced glucose intolerance.

### Lyz2Cre in the absence of palmitate infusion results in reduced absolute insulin secretion and increased insulin clearance

**Figure 2:**
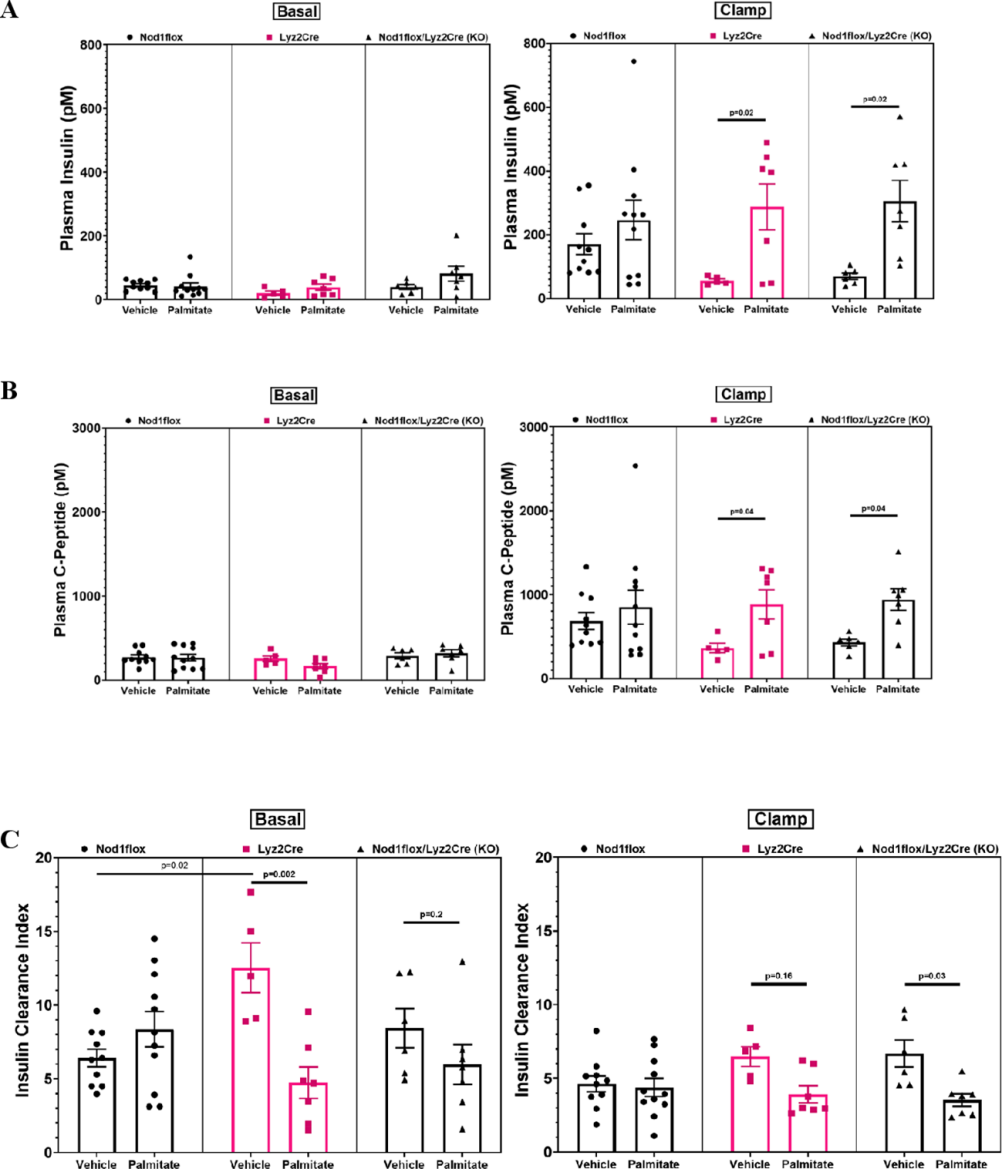
Plasma insulin **(A)**, C-peptide **(B)**, and insulin clearance index (C-peptide/Insulin) **(C)** before and during hyperglycemic clamps after a 48-h infusion of either ethanol vehicle or ethylpalmitate in 12 to 14-week-old male mice. Nod1flox: Vehicle (n=10), Palmitate (n=11); Lyz2Cre: Vehicle (n=5), Palmitate (n=7); Nod1flox/Lyz2Cre (KO): Vehicle (n=6), Palmitate (n=7). Results are mean ± SEM. One-way ANOVA: **(A)** Clamp (right panel) p=0.016; **(B)** Clamp (right panel) p=0.03; **(C)** Basal (left panel) p=0.0024, Clamp (right panel) p=0.009. Significant differences and trends (p ≤ 0.2) between groups obtained with the Holm–Sidak test are shown in the graphs.

During the clamp, plasma insulin and C-peptide levels were lower in the VEH-treated Cre and KO mice compared to the respective PAL-treated groups (**Figures 2A, B**), indicating reduced absolute insulin secretion. The insulin clearance index (C-peptide to insulin molar ratio) was significantly greater in the VEH-infused Cre mice during the basal period and tended to be greater during the steady-state clamp phase, compared to the PAL-treated Cre mice (**Figure 2C**). In the KO group, the trend was reversed, with a significantly greater insulin clearance index during the clamp phase and a tendency to increase during the basal period.

### Lyz2Cre increases insulin sensitivity in the absence of palmitate infusion and, in the presence of palmitate infusion, alleviates palmitate-induced ß-cell dysfunction

**Figure 3:**
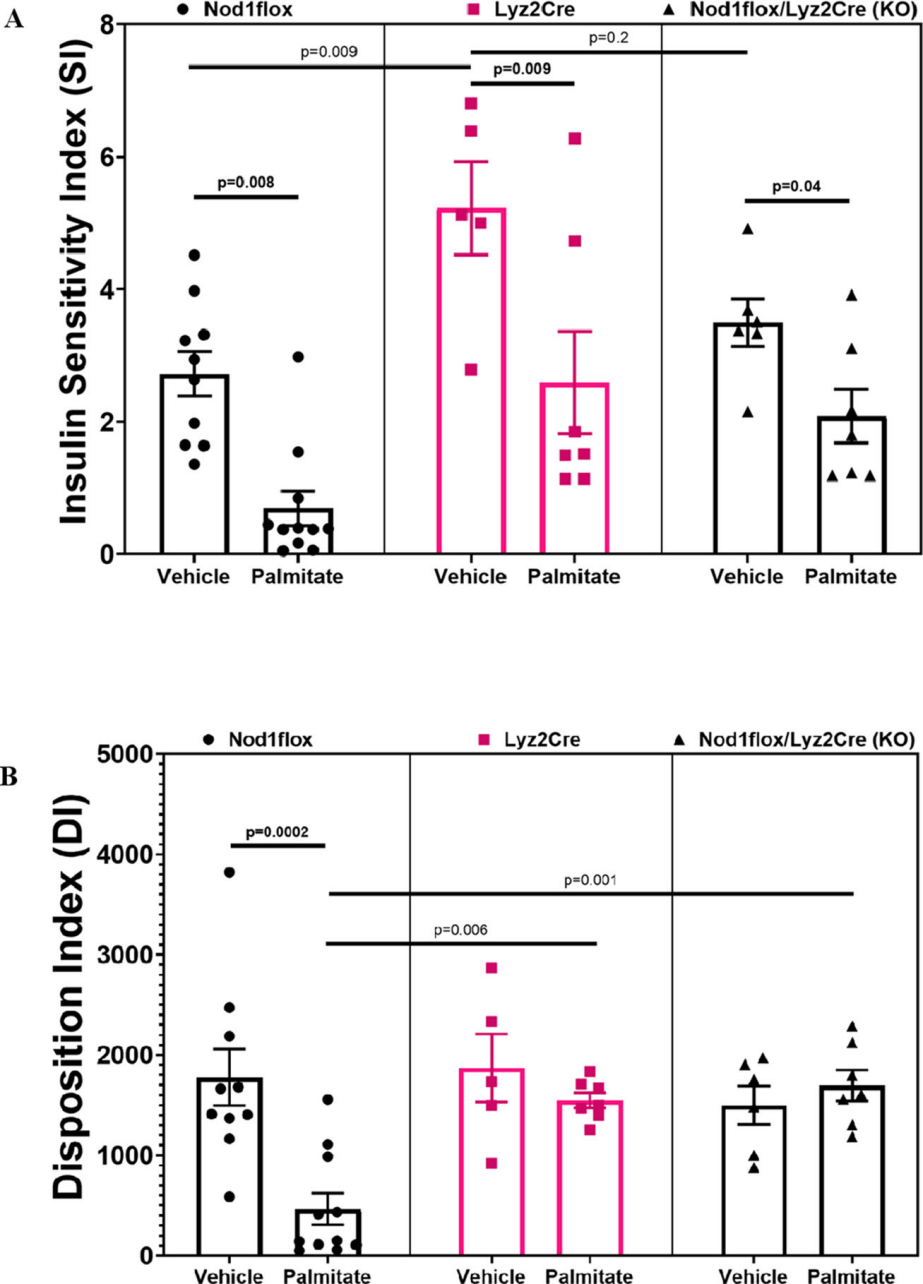
Insulin sensitivity index (SI=Ginf/Insulin, units are μmol/kg/min glucose divided by pM insulin) **(A)** and disposition index (DI=C-peptide multiplied by SI) **(B)** during hyperglycemic clamps after a 48-h infusion of either ethanol vehicle or ethylpalmitate in 12 to 14-week-old male mice. Nod1flox: Vehicle (n=10), Palmitate (n=11); Lyz2Cre: Vehicle (n=5), Palmitate (n=7); Nod1flox/Lyz2Cre (KO): Vehicle (n=6), Palmitate (n=7). Results are mean ± SEM. One-way ANOVA: **(A)** p=0.0001; **(B)** p=0.0001. Significant differences and trends (p ≤ 0.2) between groups obtained with the Holm–Sidak test are shown in the graphs.

The SI (Ginf/plasma insulin) decreased in all groups infused with PAL compared to VEH (**Figure 3A**). Although these SI results align with expectations, it is noteworthy that VEH-treated Cre mice showed higher SI compared to all other groups except the VEH-treated KO, suggesting that myeloid Cre expression in the absence of fat had an anti-inflammatory insulin-sensitizing effect. This insulin-sensitizing effect corresponded to significantly lower insulin secretion and increased insulin clearance (as described in **Figure 2**), suggesting compensatory changes in insulin secretion and clearance to maintain glucose homeostasis. In the presence of fat, however, insulin sensitivity was impaired, insulin secretion correspondingly increased, and the effect of myeloid Cre expression to protect against ß-cell lipotoxicity (anti-inflammatory effect on ß-cell function) manifested. This is evidenced by the DI, which, as expected, decreased in the PAL-treated flox control mice compared to VEH, while this decrease was prevented by myeloid Lyz2Cre expression alone or in combination with Nod1 deficiency (**Figure 3B**).

### Lyz2Cre inhibits macrophage phagocytosis

**Figure 4:**
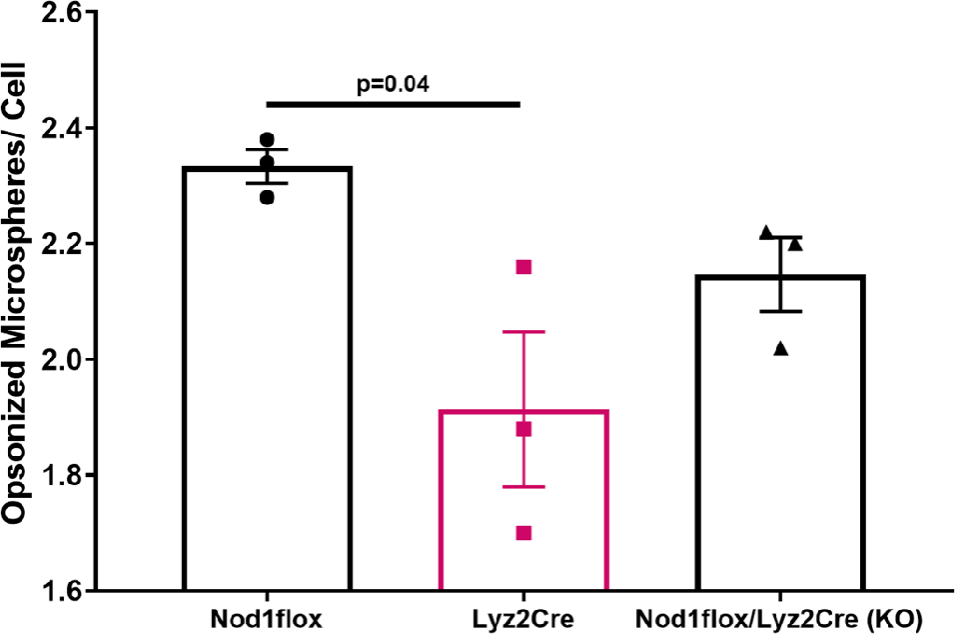
Quantitative analysis of phagocytosis in bone marrow derived macrophages of untreated Nod1 flox, Lyz2Cre, and Nod1-KO mice (n=3/group). Results are mean ± SEM. One-way ANOVA: p=0.03. Significant differences between groups obtained with the Holm–Sidak test are shown in the graph.

A phagocytosis assay with fluorescent microspheres was used to assess the effect of Lyz2Cre expression on the phagocytic ability of BMDMs. The results demonstrated a significant reduction in the phagocytic activity of BMDMs expressing Cre compared to their Cre-negative Nod1 floxed counterparts (**Figure 4**). Specifically, Lyz2Cre+ macrophages internalized significantly fewer microspheres, as evidenced by a reduced number of green-labeled (internalized) microspheres per cell (**Supplementary Figure 2A**). Although the phagocytic activity of BMDMs of Nod1-KO mice (also Cre-positive) was not significantly different from that of either Nod1 floxed or wild-type Lyz2Cre mice, by combining the two Cre-positive groups, we still observed a significant difference compared to the Cre-negative mice (**Supplementary Figure 2B**). These findings suggest that Lyz2Cre expression impairs macrophage function.

## Discussion

We herein report an unexpected effect of Lys2Cre expression in the absence of lox-P sites to protect against FFA-induced glucose intolerance. This effect could be due to a) Cre toxicity, b) haploinsufficiency of the Lys2 locus, or c) some effect of non-specific expression of Cre in distinct locations. It has been found (4) and also reported on the Jackson Lab website that insertion of Cre in the Lyz2Cre mouse deletes the endogenous gene essential for myeloid cell function as lysozyme-null mice show significantly increased mortality from airway infections (21), indicating impaired inflammatory/immune responses. In our study, even heterozygous Lyz2Cre deletion seems to yield dysfunctional myeloid cells that increase insulin sensitivity and protect against lipid-induced ß-cell dysfunction.

Most studies using the same Lyz2 Cre model as ours in glucose metabolism research did not examine the effect of Cre alone (34–41). Only one study reported the Cre-expressing mice as a separate control and demonstrated no changes in glucose tolerance evaluated with oral glucose tolerance test (OGTT) in the absence of a fat challenge (42). From our results, we surmise that Lyz2 Cre expression alone affects the function of myeloid cells, including monocytes, macrophages, and granulocytes. Given that macrophages, via their cytokine products, are the primary myeloid cells reported to be involved in insulin resistance (43, 44) and ß-cell dysfunction (26), our findings suggest an impact on macrophage function, which likely contributed to the observed changes in glucose metabolism. Indeed, we demonstrated macrophage dysfunction induced by Lyz2Cre expression, evidenced by reduced phagocytic activity. This raises the possibility that, besides glucose metabolism, other processes involving macrophage activity (for example, atherogenesis) may also be impacted. Monocytes and macrophages form a highly heterogeneous immune cell population lacking specific markers or transcriptional factors. Their gene expression patterns vary dynamically during prenatal development, adult tissue homeostasis, and inflammatory diseases (17). Current models have limitations in depletion efficiency and targeting specificity for endogenous macrophages (18). Off-target expression of Lyz2 in non-myeloid cells, such as neurons (19) that may regulate glucose metabolism (45), suggests that non-specific Cre expression in these additional cell types may contribute to the phenotype observed in our experiments. Mice exclusively carrying the Cre transgene are not always reported as controls, possibly because of the widespread belief that Cre expression has minimal to no impact on cellular function. However, our findings challenge the validity of certain conclusions in studies lacking an analysis of appropriate controls, including mice carrying the Cre transgene alone.

## Data Availability

The raw data supporting the conclusions of this article will be made available by the authors, without undue reservation.
