# Supplementary material for: Metabolic phenotypes in a Lyz2Cre recombinase mouse model
Source: Front Immunol. 2025 Mar 18;16:1499858. doi: 10.3389/fimmu.2025.1499858 (PMC11958952; doi:10.3389/fimmu.2025.1499858)
Supplement: Supplementary file 1 [file DataSheet1.pdf]

Supplementary Figure 1

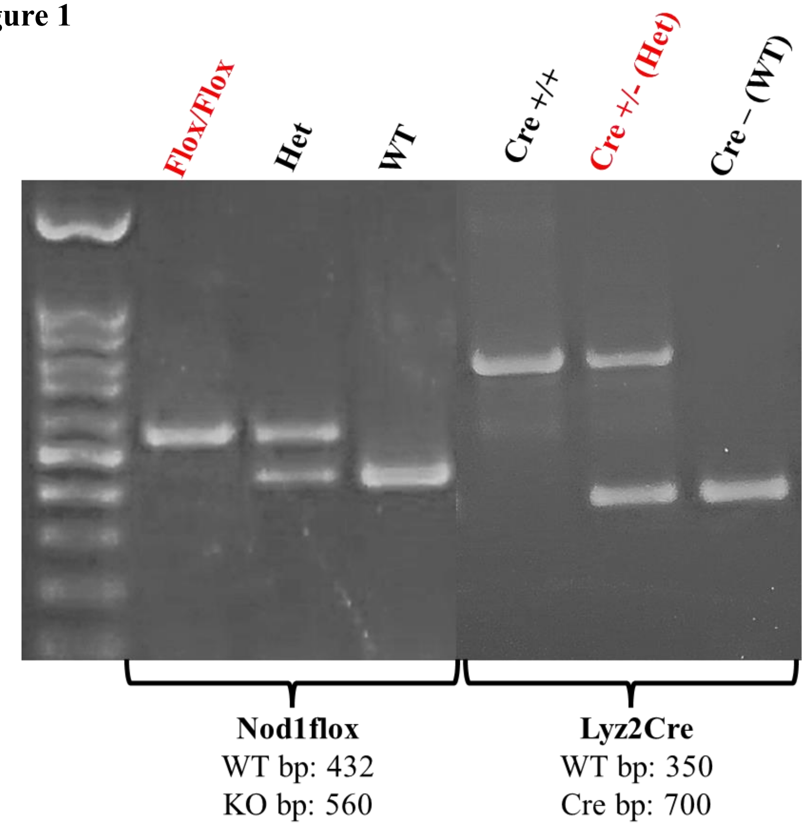

Nod1flox primer:
Forward Sequence (5’-3’): GTGAAGCCAAAGGGTCAGCA
Reverse Sequence (5’-3’): CAATGGGGTGCCTTCCATCT

Lyz2Cre primer:
oIMR3066 - CCCAGAAATGCCAGATTAGC - Mutant
oIMR3067 - CTTGGGCTGCCAGAATTTCTC - Common
oIMR3068 - TTACAGTCGGCCAGGCTGAC - Wild type

Figure S1. Mouse Genotyping Strategy. Representative agarose gel image illustrating the genotyping strategy used to identify mice carrying the WT or floxed allele and the Lyz2Cre transgene. The Nod1 primers recognize the wild type as well as the floxed gene. The Lyz2Cre primers are specific for the wild type or mutant gene or recognize both (Common).

Supplementary Figure 2

A

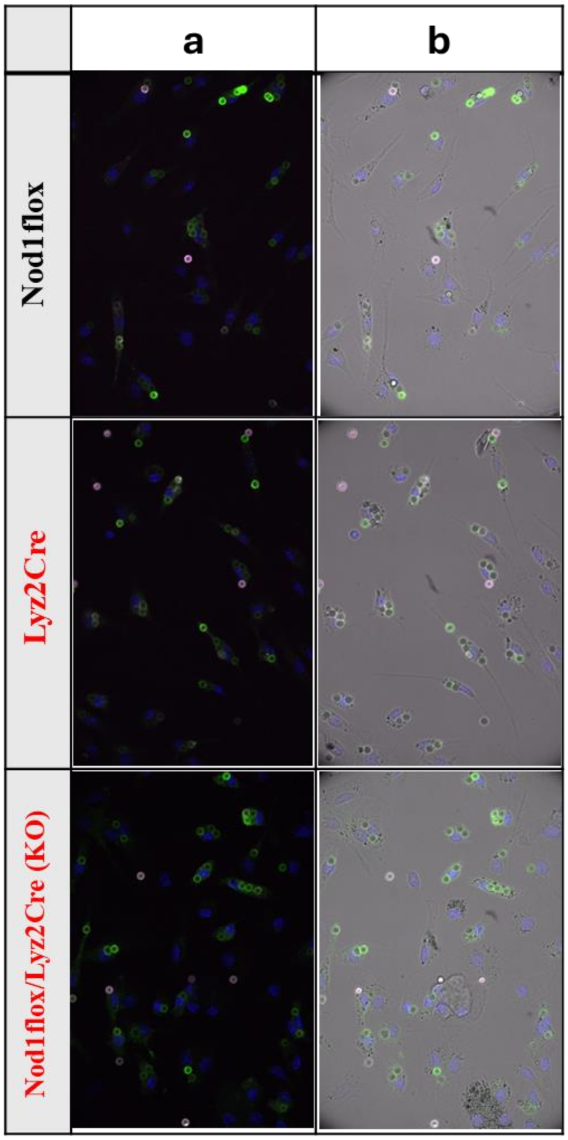

Panel **(a)**: 488-green/DAPI/ Alexa647-pink overlay.
Panel **(b)**: 488 green/DAPI/Alexa647 pink/ brightfield.
Green: phagocytosed microspheres.
Purple: external microspheres.
Blue: nuclei.

B

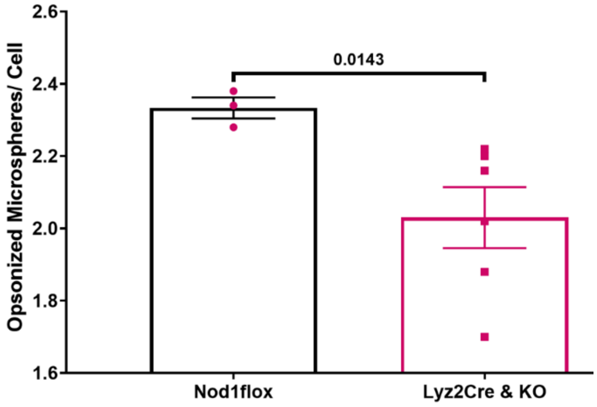

Figure S2. Phagocytosis assays in bone marrow derived macrophages. Representative confocal images showing phagocytosed (green) and external (purple) microspheres **(A)**, quantitative analysis of phagocytosis **(B)**. n=3-6/group. Results are mean ± SEM. Student's t test; p=0.0143 vs Nod1flox.
